# Spontaneous echo contrast masking thrombus in giant left atrium of mitral stenosis-a dilemma in clinical diagnosis

**DOI:** 10.12669/pjms.315.7602

**Published:** 2015

**Authors:** AKM Monwarul Islam, Md. Abdus Salam, Md. Zahidus Sayeed, Md. Golam Kibria

**Affiliations:** 1 AKM Monwarul Islam, MD, FRCP. Assistant Professor, Department of Cardiology, Jessore Medical College, Jessore 7400, Bangladesh; 2 Md. Abdus Salam, PhD, FRCP. Associate Professor, Department of Microbiology, Rajshahi Medical College, Rajshahi-6000, Bangladesh; 3 Md. Zahidus Sayeed, MD. Consultant Cardiologist, Department of Cardiology, Rajshahi Medical College, Rajshahi, Bangladesh; 4 Md. Golam Kibria, MS. Associate Professor, Department of Cardiovascular and Thoracic Surgery, National Institute of Cardiovascular Diseases, Dhaka, Bangladesh

**Keywords:** Mitral stenosis, Left atrium, Thrombus, Transesophageal echocardiography

## Abstract

Spontaneous echo contrast (SEC) and thrombus in enlarged left atrium (LA) are common in mitral valvular disease (MVD) and SEC is considered to be a prethrombotic condition. Reliable exclusion of LA thrombus is important before any definitive curative attempts like percutaneous transluminal mitral commissurotomy (PTMC), closed mitral commissurotomy (CMC) or innovative therapies like pulmonary vein isolation and percutaneous closure of the LA appendage. Echocardiography, particularly the transesophageal echocardiography (TEE) is considered to be the gold standard for the diagnosis and to exclude LA thrombus. However, LA thrombus may remain rarely undetected even by TEE potentially making the interventions a risky job. We present a case of mitral stenosis (MS) with giant LA where profuse, dense SEC masked the underlying thrombus in the LA cavity.

## INTRODUCTION

Spontaneous echo contrast (SEC) and thrombus in enlarged left atrium (LA) are common in mitral valvular disease (MVD). Presence of SEC is the most evident sign of slackened blood flow^1^ and is associated with an increased risk for thromboembolism.^2^ However, when present in the LA, thrombus, but not SEC, is a contraindication for percutaneous transluminal mitral commissurotomy (PTMC) and closed mitral commissurotomy (CMC). Reliable exclusion of LA thrombus is also important before other therapeutic modalities like pulmonary vein isolation and percutaneous closure of the left atrial appendage (LAA).

Echocardiography, particularly the transesophageal echocardiography (TEE) can efficiently diagnose and exclude LA thrombus and is regarded as the gold standard in this regard.^1^ However, occasionally even by TEE, LA thrombus may remain undetected, potentially raising the periinterventional risk disproportionately.

The present case of mitral stenosis (MS) with giant LA where profuse, dense SEC masked the underlying thrombus in the LA cavity is being reported primarily to share the experience of such atypical clinical condition and to disseminate the limitation of echocardiography.

## CASE PRESENTATION

**Fig.1A:**
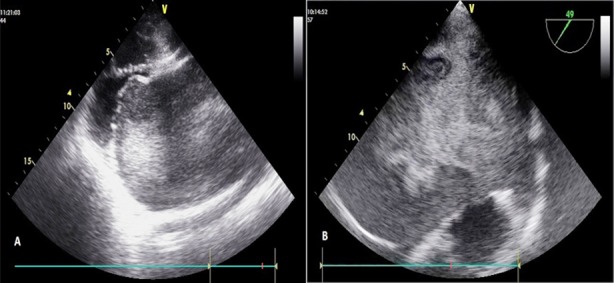
A. Transthoracic echocardiography left parasternal long axis view showing features of severe mitral stenosis, dilated left atrium and dense, spontaneous echo contrast. B. Transesophageal echocardiography midesophageal view at 49° showing dilated left atrium, dense, spontaneous echo contrast, and appendage free of definite thrombus.

A 26-year-old lady presented with shortness of breath and cough for 6 months. On examination, 1^st^ heart sound was of variable intensity and there was a localized, low-pitched, mid diastolic murmur (grade 4/6), more pronounced at apical area in left lateral position. ECG showed atrial fibrillation (AF) and right ventricular hypertrophy. Transthoracic echocardiography (TTE) revealed features of severe MS, giant LA measuring 100 mm with profuse, dense SEC in LA, but no definite thrombus. Valve morphology was otherwise suitable for PTMC (Fig.1A).

Transesophageal echocardiography (TEE) was done which also showed similar findings, again without any definite thrombus (Fig.1B).

**Fig.2:**
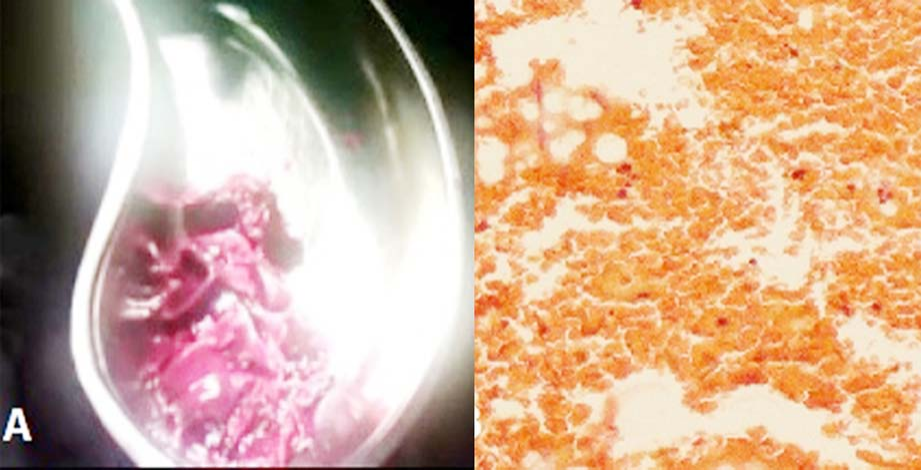
A. Thrombus extracted from left atrium during open mitral commissurotomy. B. Histopathology of the extracted material showing features of thrombus.

She was diagnosed as a case of severe MS, pulmonary hypertension, AF and SEC in the LA. Regarding management, PTMC was thought initially but apprehension of probable technical difficulties in association with hugely dilated LA, it was abandoned. Eventually open mitral commissurotomy (OMC) was done and during operation a large amount of thrombus was found in LA cavity (Fig.2A), which was later confirmed by histopathology (Fig.2B).

## DISCUSSION

Severe MS is commonly associated with both SEC and thrombus in dilated LA. The LAA is the most common site of thrombus formation because of sluggish flow, especially when accompanied by AF. SEC, also known as ‘*smoke’*, is a swirling pattern of increased echogenicity, distinct from white noise artifacts in the LA or LAA caused by ultrasonic backscatter from red blood cell aggregates seen in blood stasis or low-velocity blood flow.^2^ Atrial fibrillation, larger LA diameter and LAA area, lower LAA blood flow velocity, smaller MV area and absence of significant mitral regurgitation are all considered to be the predisposing factors for SEC.^3^

The clinical importance of SEC is its association with LA thrombus, increased thromboembolic complications and death,^2^ although SEC is not always accompanied by LA thrombus and thrombus may be formed in absence of detectable SEC as well. The prevalence of thrombus and SEC in LA varies according to the clinical conditions. LA SEC was observed in approximately 65% of MS patients undergoing TEE.^4^ In a study involving 100 patients with severe MS and AF, 38% had LA thrombi, whereas LA SEC was found in 54% of patients.^5^

In the present case, giant LA and presence of AF predisposed to stasis of flow of blood in LA favouring SEC and thrombus formation. Proper identification of SEC and LA thrombus is of crucial importance for both diagnostic and therapeutic purposes; early cardioversion is avoided in presence of LA thrombus and appropriate anticoagulant therapy is instituted. Further, presence of LA thrombus is a contraindication for PTMC and CMC, the usual practice is to administer anticoagulants for 6 to 12 weeks and interventional treatment is adopted only after reevaluation by echo. For identification of LA thrombus, a number of tests are available. TTE and TEE are the commonly used modalities. The sensitivity of TTE for detection of LA thrombus is modest, at best, i.e. 59-63%, though specificity is high (95-99%).^6^ On the other hand, TEE is the gold standard test for diagnosing LA thrombus with almost 100% sensitivity and specificity.^6^

However, there are several pitfalls in exclusion of thrombus in LA by TEE e.g. multi-lobed structure of LAA, pectinate muscles within the LAA wall and a reverberation artifact originating from the ridge at the mouth of the left upper pulmonary vein (*Coumadin ridge*).^7^ Sometimes, it becomes impossible to differentiate dense SEC from underlying thrombus and in the present case, profuse, dense SEC with its whirling motion created difficulties in diagnosing the thrombus in the LA cavity, presumably due to near identical radio-density. Inter-observer variability in interpretation is another possibility; however, the echocardiography of the present case was scrutinized by two efficient echocardiographers, so, chance of inter-observer variability was less. Considering the fact, PTMC in this particular case could have been a risky job with a potential of systemic thromboembolism including stroke. A disadvantage of both two-dimensional (2D) TTE and TEE is that they provide only a thin slice or section of cardiac structures at any given time limiting their utility in comprehensively assessing the LAA for thrombus.^8^ On the other hand, real time (RT) three-dimensional (3D) TTE^9^ and TEE can encompass whole of the LAA in its dimensions in the acquired data set, which can then be cropped and sectioned systematically at any desired angulation to more definitively look for clot.^8^

In comparison to 2D, 3D echo can more efficiently differentiate a clot from pectinate muscles in the LAA, which can mimic a thrombus resulting in patient mismanagement.^8^ Because of very limited availability of 3D echo in Bangladesh, evaluation by 3D echo was not done in the case presented here. Recently, because of semi-invasive nature of TEE, reliable alternative modalities are being sought. In this regard, cardiac computed tomography (CT)^9^ and cardiac magnetic resonance (CMR)^10^ have been reported to be reliable alternatives to TEE with high sensitivity for diagnosing LA thrombus. A recent systematic review^9^ revealed cardiac CT, particularly when delayed imaging is performed, to be a reliable alternative to TEE for the detection of LA thrombi avoiding the discomfort and risks associated with TEE. The mean sensitivity and specificity were 96% and 92% and for delayed imaging, it was 100% and 99%, respectively. The major advantages of CMR are the absence of radiation exposure, the high temporal and spatial resolution and the ability to characterize the composition of the tissue which allows a detailed assessment of the structure and function of the LA; however several disadvantages are also evident, such as cost, limited availability, incompatibility of certain prosthetic materials and time consuming analysis. Recently, CMR was found to be a reliable alternative to TEE for complete noninvasive evaluation of LAA thrombus and pulmonary vein anatomy in patients with AF before pulmonary vein isolation without obligate need for TEE.^10^

## CONCLUSION

In general echocardiography is sufficient to diagnose and exclude the LA thrombus in clinical practice. Sometimes, SEC can make this otherwise straightforward job difficult, even TEE cannot rule out thrombus but should lead to the verbalization of a suspicion with the need for further imaging. Physicians should keep in mind the limitations of echocardiography, though uncommon, in diagnosing LA thrombus in presence of dense SEC and have appropriate preparedness to deal with the situation.
